# Travis County Medical Society

**Published:** 1885-09

**Authors:** 


At the last regular meeting of the Travis County (Tex.) Medical Association—it being the occasion of the semi-annual election of officers—the following were elected: President, Dr. F. E. Daniel, of Austin; Vice-President, Dr. Dean, of Hornby; Secretary, Dr. Ralph Steiner; Treasurer, Dr. T. J. Bennett. This active Association is doing good work and extending its membership. Dr. G. W. Christian, a prominent physician of Burnet, Burnet county, was admitted to membership. It is proposed to create a district Association, embracing Travis, Burnet, Mason and one or two other adjoining counties, shortly.

